# Application of E-coach chronic disease management model in rehabilitation management of patients with arteriosclerosis obliterans

**DOI:** 10.1186/s41043-023-00454-7

**Published:** 2023-10-27

**Authors:** You-Yuan Yuan, Wen-Dong Cao, Xiao-Hong Zhang, Rong-Xin Du, Xue-Qi Wang, Jing Li, Juan Chen, Jun-Zi Yang, Jia-Qi Chen

**Affiliations:** 1 Department of Interventional Therapy for Tumor and Vascular Disease, Shanxi Bethune Hospital, Shanxi Academy of Medical Sciences, No. 99 of Longcheng Street, Xiaodian District, Taiyuan, 030032 China; 2 Department of Nursing, Shanxi Bethune Hospital, Shanxi Academy of Medical Sciences, Taiyuan, 030032 China; 3 Department of Urology Surgical, Shanxi Bethune Hospital, Shanxi Academy of Medical Sciences, Taiyuan, 030032 China

**Keywords:** Chronic disease management mode, Health coaching technology, Continuous nursing, Arteriosclerosis obliterans

## Abstract

**Objective:**

To explore the effect of a health (E)-coach chronic disease management model on the rehabilitation behaviour management of patients with arteriosclerosis obliterans (ASO).

**Methods:**

The E-coach chronic disease management model was constructed based on a literature review and expert interviews. The effect of the E-coach model on patients with ASO during hospitalisation was analysed by comparing the compliance rates of blood glucose control, blood pressure control, drug compliance, ankle-brachial index, 6-min walking test (6MWT) and pain-free walking distance (PFWD) scores between the E-coach and control groups.

**Results:**

In total, 212 patients with ASO were included in this study. After the intervention, the blood pressure compliance rate (44.8% vs. 65.7%) and blood glucose compliance rate (48.6% vs. 66.8%) were higher in the E-coach group than in the control group (*p* < 0.05). After intervention, compared with the control group, the patients in the E-coach group had better drug compliance (6.8 ± 1.9 vs. 7.9 ± 1.0), and the difference was statistically significant (*p* < 0.05). The scores for the 6MWT (329.19 ± 5.58 vs. 353.00 ± 9.76; 412.65 ± 12.59 vs. 499.16 ± 18.43) and PFWD (219.15 ± 11.96 vs. 225.36 ± 16.13; 331.62 ± 51.36 vs. 369.42 ± 75.71) tests were significantly higher in the E-coach group than in the control group at 1 and 6 months after intervention (*p* < 0.05).

**Conclusion:**

The E-coach chronic disease management model can effectively improve the control rates of blood glucose and blood pressure and the behaviour management of patients with ASO and is thus worthy of clinical reference.

## Introduction

The prevalence of lower extremity arteriosclerosis obliterans (ASO) is more than 10%, second only to coronary heart disease and stroke [1–3]. ASO is the main cause of lower limb amputation, accounting for 40~60% of all amputation patients [4]. There is even a risk of death. Timely detection and control of risk factors leading to atherosclerosis is the key to delaying the disease process [5]

The low level of primary health service in China and the difficulty of seeing a doctor in tertiary hospitals have led to division, discontinuity and poor ASO management compliance. In fact, there is no unified standard for the management of patients with lower extremity ASO in China, and most approaches remain in the exploratory stage [2]. In recent years, the health coaching (E-coach) approach has been widely used and studied for the management of chronic diseases. At present, studies both at home and abroad have confirmed certain shortcomings in health coaching technology [6, 7], including the long intervention cycles, the inability to participate in remote areas and high intervention costs. The application of the E-coach method in the management of hypertensive patients has achieved good results in terms of the improvement in the self-management, medication compliance and clinical indicators of hypertensive patients [8]. The internet is widely used in chronic disease management and can provide real-time monitoring and tracking, telemedicine services, two-way referral, accurate appointments, intelligent consultation and online drug consultation services [9, 10]. In this study, traditional health coaching and internet technology were integrated to form an E-coach chronic disease management modelGuided by the theory of behaviour change, and following the process of chronic disease coach, an empirical study on the management of ASO patients has achieved good results and provided a basis for its popularization and application. At the same time, it can help nursing staff to provide new ideas for family nursing and continuous nursing of lower extremity arteriosclerosis obliterans.

## Construction of E-coach chronic disease management model

### Preliminary development of content framework

The China National Knowledge Infrastructure, Wanfang Data, China Science and Technology Journal Database, PubMed, Web of Science, and ClinicalKey Nursing English databases were selected for the search, with the search period set from 1 January 1990 to the present date. Keywords were used for the search, with the Chinese keywords including the terms for ‘health coaching technology’, ‘chronic disease management’, ‘telemedicine’ and ‘mobile medicine’, and the English keywords including ‘Health coaching’, ‘chronic disease management’, ‘mHealth’ and ‘teleHealth’. The current research situation and existing problems in the field of health coaching technology and chronic disease management were identified through a literature review, and the structural framework of the E-coach model was built.

### Expert interview

Using the focus group interview method, experts in related fields were invited to evaluate and revise the E-coach structural framework.

#### Set-up of an expert group

The team members included ten experts operating in the fields of medical treatment (*n* = 3), nursing (*n* = 5), epidemiology (*n* = 1) and public medical and health management (*n* = 1). The selection criteria included: (1) those with associate professor titles or above, or intermediate or above, and who had obtained a master’s degree; (2) those who engaged in related professional work for more than eight years, with rich theoretical knowledge and management practice experience; and (3) those who had high enthusiasm for this study and a willingness to participate.

#### Interview and data analysis

The interviews were conducted at the shift office, with the researcher serving as the host alongside one graduate student from the medical department and one from the nursing department. All interviews were recorded with the consent of the interviewees. The interview outline was as follows: (1) Is the framework structure of the E-coach chronic disease management model comprehensive and applicable? Do you have any other opinions? (2) What are the implementation steps, process objectives, and methods of the management mode? (3) Your opinions and suggestions for the E-coach chronic disease management model. The interview lasted 45 min. The researcher built relevant content on the management mode around the interview outline and encouraged the experts to fully express their opinions. After the interviews, the two graduate students exchanged the results of the audit records and compared them with the recordings when there were disputes. Based on the interview records, the members of the research team transcribed the recordings and analysed the data using Giorgi’s method of analysis.

Preliminary results were obtained from the first round of interviews, and the supporting education system proposed by Orem was selected as the theoretical framework. The nursing procedure was used as the basic tool, and the decomposition steps and methods of the model were constructed.

The second round of interviews was conducted based on the analysis of the first round. The outline was as follows: (1) Whether the current management mode is comprehensive, whether it is supplemented or modified; and (2) opinions or suggestions regarding the E-coach model.

### Determination of the E-coach chronic disease management model

#### Forming the schema

**Fig. 1:**
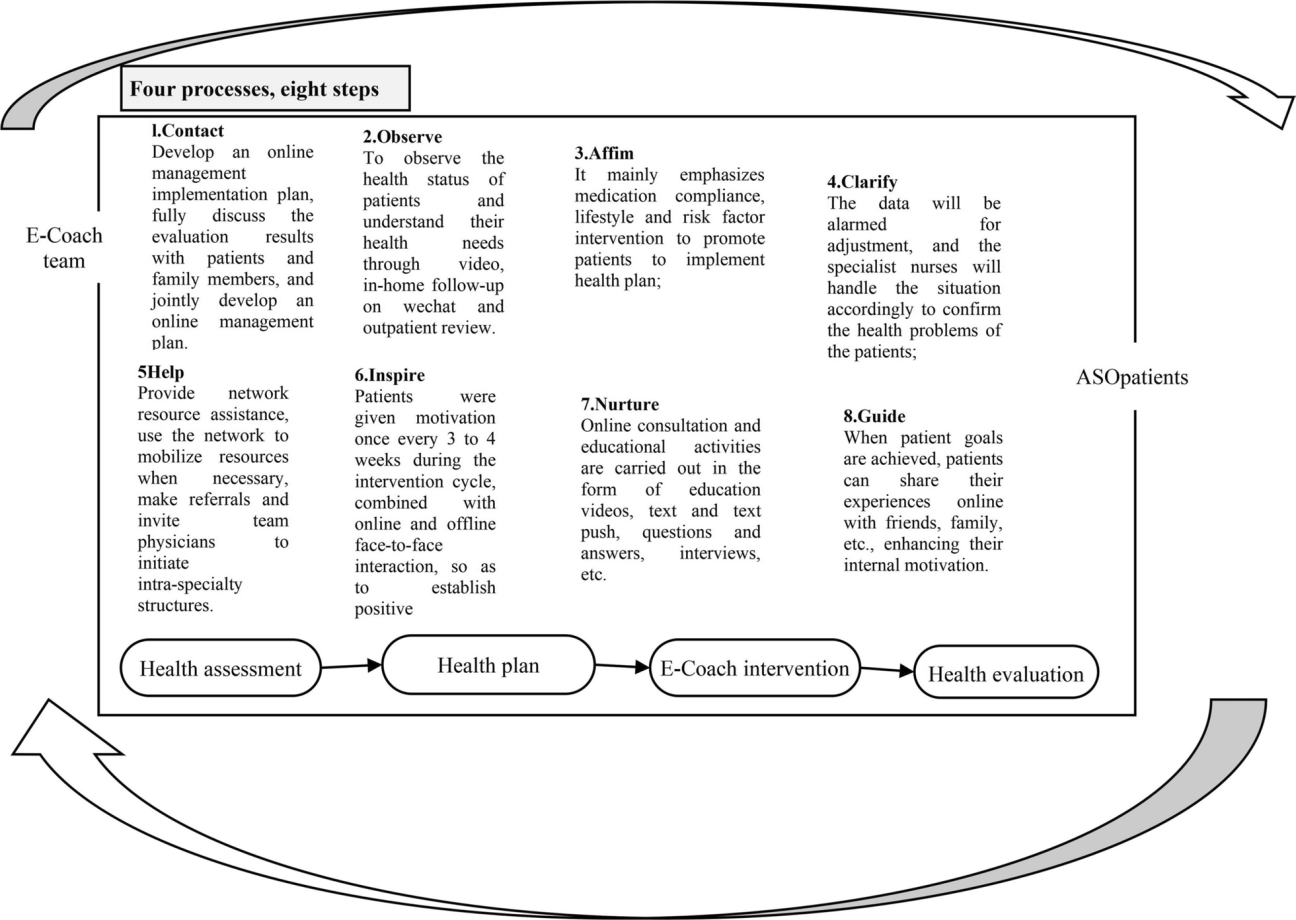
Eight steps of ‘‘COACHING’

Based on information from the WeChat official account and wjx.cn, data collection was carried out, and the organisational framework was constructed by following the eight steps of ‘coaching’ and combining various nursing procedures (Fig. 1).

#### E-coach team structure and members’ responsibilities

The team members included vascular specialists, coaching nurses, rehabilitation teachers, nutritionists and other multidisciplinary members. Each member had > 5 years of clinical work experience and had obtained a coaching nurse or specialist nurse certificate in health management. In terms of member duties, specialists are responsible for the diagnosis and treatment plan of patients with ASO, nurses are responsible for health assessment, rehabilitation plans and E-coach intervention and evaluation, as well as organisation and coordination of team services, and nutritionists and rehabilitators are responsible for responding to health needs and providing treatment and health education related to their fields when referrals occur.

#### Implementation steps, objectives and methods of the E-coach chronic disease management model

The implementation steps, objectives and methods of the E-coach model were finally formed according to the eight steps in the framework combined with the concept of Internet + (Table 1).

**Table 1:** Implementation steps, objectives and methods of E-Coach chronic disease management model

Steps	Objectives	Implementation method
1. Contact	Establish a good communication channel with patients, and regularly grasp the health status and needs of patients	Ask the patient to follow the WeChat official account of ‘Bai Qiu En Qian Yi Ren’ to regularly check the pushed video, fully discuss the evaluation results with the patients and their families, and formulate a health education plan in combination with the actual situation of the patients’ families;
2. Observe	Understand the health needs of patients	Instruct patients to fill in the data of wjx.cn, and formulate the time and method of follow-up through the observation of the data trend line, including video, door-to-door, WeChat and outpatient review;
3. Strengthen	Stress drug compliance, lifestyle, exercise and risk factors intervention to promote patients to implement health plans	(1) Urge patients to upload daily heart rate, blood pressure and blood sugar values (the frequency is determined according to the patient's evaluation results); (2) Fill in the amount of exercise on the day; (3) Exercise scheme: ① Buerger exercise. Ask the patient to lie on his back, raise his lower limbs by 45° for 1 ~ 2 min. In the sitting position, the feet droop naturally, and the combined actions of back extension, plantar flexion and circling are performed for 3 ~ 5 min; lay his lower limbs flat and rest for 2 ~ 5 min. Repeat the above actions continuously for 3 ~ 5 times, ② Fast walking training + Buerger exercise. Inform the patient to walk at the fastest speed on flat ground or a corridor with a length of 30 m to reach moderate to severe claudication level, then rest properly, resume walking after the pain disappears, and record the walking distance in this cycle. The training time is 30 min, and the training time is 3 times a week, step by step according to the patient’s tolerance. ③ Lower limb resistance training + fast walking training + Buerger exercise. Elastic belt training is used for lower limb resistance training, including five movements: hip walking lunge, knee lifting, standing side kicking, flexing feet back training and sitting leg kicking. Each movement is carried out in 2 ~ 3 times for 10 ~ 20 times, and the left and right legs are alternated, with a rest of 1 ~ 2 min between every two movements and training 3 ~ 5 times a week
4. Clarify	Ensure the effectiveness and feasibility of the implementation of measures	According to the patient’s data detection trend chart, if it exceeds or exceeds the critical detection line, the specialist nurse will make adjustments according to the situation, such as guiding observation, suggesting medical treatment, WeChat/telephone/face-to-face, etc., and confirm the patient’s health status and problems;
5. Help	Provide patient self-management support	Provide network resource help, and specialist nurses assess the patient's conditions and needs. If necessary, use the network to mobilize resources, refer and invite team doctors to start structured assessment;
6. Encourage	Ensure the sustainability of patient cooperation	Give patients motivation intervention cycle, once every 3 ~ 4 weeks, combined with online and offline face-to-face interaction, to establish positive experience and give patients motivation;
7. Educate	Provide education and support in knowledge and skills	Carry out media education in the form of education videos, graphic push, Q&As, interviews, etc., and conduct online consultation and education activities in combination with the needs of patients;
8. Guide	Increase patients’ sense of accomplishment and integrate health goals into daily life	When the patient’s goal is achieved, the patient can share his experience with other patients and their families online to enhance his internal motivation

## Application of E-coach chronic disease management model

### Participants

**Fig. 2:**
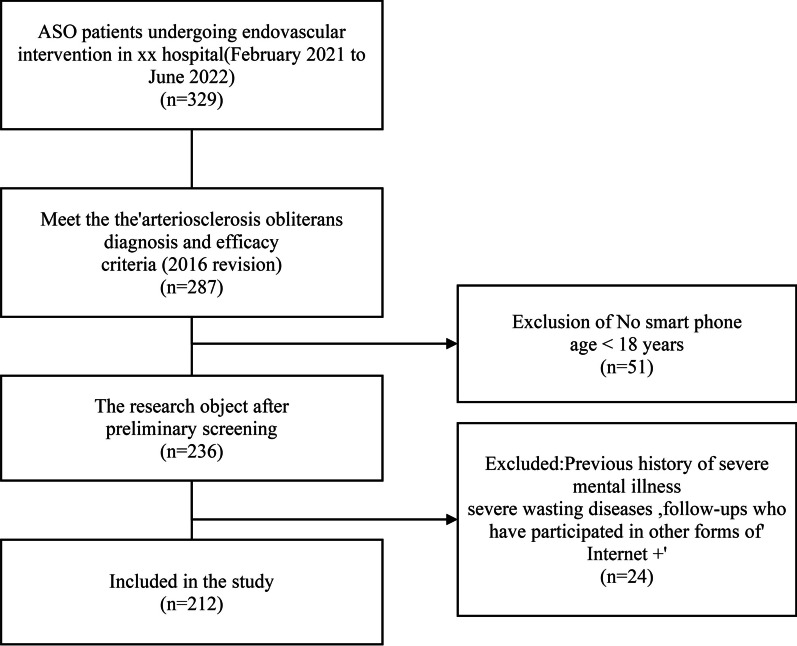
Inclusion process of research objects

A non-concurrent case–control study was conducted. ASO patients who underwent endovascular interventional therapy for the first time in Shanxi Bethune Hospital from February 2021 to July 2021 were selected as the control group (*n* = 101), and ASO patients who underwent endovascular interventional therapy for the first time in the same hospital from February 2022 to July 2022 were selected as the E-coach group (*n* = 111). The study was included according to the 15% loss of follow-up rate. The inclusion criteria were as follows: (1) aged ≥ 18 years; (2) patients meeting the criteria for the diagnosis of ASO (revised draft in 2016) [11]; (3) patients or family members able to use smartphones and read internet-based information; and (4) patients providing informed consent. The exclusion criteria included: (1) patients suffering from moderate and severe cognitive dysfunction and mental illness and who could not participate in self-management; (2) patients who suffered complications with severe organic heart disease, malignant tumour, liver and kidney failure, and other diseases; (3) patients who had participated in other forms of ‘Internet+’. The inclusion process used for the research participants is shown in Fig. 2. Informed consent was obtained from the patients or their families and the study passed the ethics examination and received approval from the hospital.

### Interventions

#### Health management of the control group

Specialised health education for patients with ASO includes: (1) introducing the aetiology, clinical manifestations, treatment and nursing points of the disease; (2) explaining the time, methods and precautions of exercise during the peri-operation period; (3) explaining the names, effects and adverse reactions of drugs for oral and intravenous treatment; (4) explaining the requirements and significance of special examination cooperation; (5) detailing the evaluation methods for the pain and the side effects of drugs; (6) outlining the key points of ankle pump exercise and Buerger exercise post-surgery; and (7) detailing the education after discharge. Patients were followed up by telephone regularly at 1, 3, and 6 months after discharge, including functional exercise, diet, sleep, and psychological state; the outpatient clinic was notified to follow-up, and three folding pages formulated by the department were distributed to patients to explain the manifestations and prevention of complications and teach patients to identify complications early.

#### E-coach group

Based on the ASO routine management mode, the E-coach chronic disease management mode, which involved a multidisciplinary team led by coaches and nurses, was implemented for 6 months.

### Evaluation indicators

#### Compliance rate of blood pressure and blood sugar

Blood pressure compliance means that the patient’s blood pressure is controlled within the diagnosis and treatment standard of < 140/90 mmHg > , and the blood pressure compliance rate is the number of patients with blood pressure compliance/total number of patients under management × 100%. Six months before and after the intervention, the patient was asked to rest for 30 min in the supine position, and the nurse measured the value using the same electronic sphygmomanometer. According to the control target of the Guidelines for the Prevention and Treatment of Type 2 Diabetes Mellitus in China-2017 Edition [12], the blood glucose compliance rate should be 4.4 mmol/l < fasting blood glucose < 7.0 mmol/l, and the blood glucose compliance rate is the number of patients with blood pressure compliance/total number of patients under management × 100%.

#### Drug compliance

The Morisky drug compliance scale, which consists of eight items, was used for the evaluation. In terms of scoring method, if ‘yes’ is selected for items 1–4 and 6–7, the score is 0, while if ‘no’ is selected, the score is 1; item 5 is scored in reverse, while item 8 is scored using the 5-point Likert scoring method. The sum of the scores for each item is the total score; 8 points indicates good compliance, 6–8 points indicates medium compliance, and < 6 points indicates poor compliance. Cronbach’s alpha coefficient and the test–retest reliability coefficient of the Chinese version of the scale [13] were 0.736 and 0.944, respectively. The scale was evaluated by researchers who were trained in data collection and who did not participate in the intervention activities when they met the patients at the outpatient clinic.

#### Ankle-brachial index

The recovery effect was evaluated using a pain assessment and the ankle-brachial index (ABI), with the time nodes preoperative, 3 days after surgery, and 6 months after surgery.

#### Six-minute walking and pain-free walking distance tests

The 6-min walking test (6MWT) was used to evaluate the exercise tolerance and limb rehabilitation level of the patients in the out-of-hospital rehabilitation stage, and the pain-free walking distance (PFWD) test was used to evaluate the patients’ walking impairment [14]. The patients were asked to walk at the fastest tolerable speed for 6 min. When they felt tired, they could slow down or stop to rest before continuing the walking after recovery. The PFWD result, that is, the distance from walking to numbness and pain of the lower limbs, was recorded, and the total walking distance of the patients in 6 min was recorded to obtain the 6MWT results. The 6MWD and PFWD were measured before operation, 3 days and 6 months after operation.

### Statistical analysis

The data were analysed using SPSS22.0 software. The mean ± standard deviation was used for measurement data conforming to a normal distribution, and the *t* test was used for inter-group comparisons. Metrical data that did not conform to a normal distribution were described by median and quartile spacing, with the Mann–Whitney *U* test employed for the inter-group comparisons. The counting data were expressed in terms of frequency and composition ratio, and the comparison between groups was performed using the chi-squared (*χ*^2^) test or Fisher’s exact probability method. Statistical significance was set at *p* < 0.05.

## Results

### Comparison of two sets of general data

Of the 212 patients with ASO included in this study, 204 completed the follow-up and eight were lost to the follow-up (five in the control group and three in the E-coach group); the lost follow-up rates were 5% and 3%, respectively. There was no significant difference in the general data between the two groups (*p* > 0.05) (Table 2).

**Table 2:** Comparison of general data between two groups of patients

Indicators	Control group *n* = 96	E-coach group *n* = 108	*t*/*Z*/χ^2^	*P*
Gender (Person%)			0.023	0.889
Male	48 (50.0)	61 (56.5)		
Female	48 (50.0)	47 (43.5)		
$\overline{x} \pm s$ age	65.3 ± 12.6	66.1 ± 14.3	0.567	0.658
$\overline{x} \pm s$ course of disease	3.35 ± 2.26	4.11 ± 2.48	0.136	0.894
Medical expenses (per person %)			–	0.865
Medical insurance for employees	53 (55.2)	61 (56.5)		
Resident medical insurance	29 (30.2)	29 (26.9)		
Others	14 (14.6)	11 (10.2)		
None	0 (0)	7 (6.4)		
Education level (person%)			− 0.159	0.969
Primary school and below	11 (11.5)	19 (17.6)	− 0.274	0.759
Junior/senior high school	43 (44.7)	45 (41.7)		
Junior/undergraduate	26 (27.1)	24 (22.2)		
Master degree or above	16 (16.7)	20 (18.5)		
Complicated with chronic diseases (person%)			− 0.315	0.753
None	16 (16.7)	14 (13.0)		
1 kind	31 (32.3)	22 (20.3)		
2 kinds and above	49 (51.0)	72 (66.7)		
Combined medication (person%)			− 0.111	0.912
None	9 (9.4)	15 (13.9)		
≤ 3 kinds	36 (37.5)	45 (41.7)		
≥ 4 kinds	51 (53.1)	48 (44.4)		
Rutherford levels (%)			0.861	0.605
Level 3	18 (18.7)	28 (25.9)		
Level 4	42 (43.8)	45 (41.7)		
Level 5	36 (37.5)	35 (32.4)		

### Comparison of blood pressure and blood glucose before and after intervention and compliance rate

Before the intervention, there were no significant differences in blood pressure, fasting blood glucose or compliance rates between the two groups (*p* > 0.05). After the intervention, the compliance rate of blood pressure in the E-coach group increased from 36.5 to 65.7%, while that in the control group increased from 31.2 to 44.8%, and the compliance rate of blood glucose increased from 26.3 to 66.8% in the E-coach group and from 27.5 to 48.6% in the control group. After the intervention, the blood pressure compliance rate (44.8 vs. 65.7%) and blood glucose compliance rate (48.6 vs. 66.8%) were higher in the E-coach group than in the control higher (*p* < 0.05) (Tables 3 and 4).

**Table 3:** Comparison of blood pressure between the two groups before and after intervention

Group	*n*	$\overline{x} \pm s$ systolic blood pressure		$\overline{x} \pm s$ diastolic pressure		Blood pressure compliance rate%	
		Before intervention	After intervention	Before intervention	After intervention	Before intervention	After intervention
Control group	96	142.6 ± 16.5	139.6 ± 14.5	91.5 ± 9.5	80.6 ± 11.1	31.2	44.8
E-coach group	108	152.6 ± 16.5	132.6 ± 6.5	89.3 ± 10.5	75.6 ± 8.7	36.5	65.7
*t*/*χ*^2^		− 0.093	− 2.316	0.815	1.486	0.073	4.617
*P*		0.786	0.013	0.442	0.039	0.819	0.012

**Table 4:** Comparison of blood glucose between the two groups before and after intervention

Group	*n*	$\overline{x} \pm s$ fasting blood glucose		$\overline{x} \pm s$ glycosylated haemoglobin		Blood glucose compliance rate%	
		Before intervention	After intervention	Before intervention	After intervention	Before intervention	After intervention
Control group	96	9.72 ± 3.49	8.35 ± 3.10	8.20 ± 1.78	7.50 ± 1.69	27.5	48.6
E-coach group	108	8.71 ± 3.31	8.10 ± 2.54	8.24 ± 3.49	6.43 ± 1.42	26.3	66.8
*t*/*χ*^2^		0.096	3.315	− 0.099	0.413	0.021	5.716
*P*		0.214	0.023	0.854	0.011	0.878	0.017

### Comparison of drug compliance and ankle-brachial index between the two groups before and after intervention

After the intervention, 55.6% of the patients in the E-coach group and 33.3% in the control group rated as ‘good’, with the score base increasing by 2.3 ± 1.7 in the E-coach group and 1.5 ± 1.2 in the control group after the intervention. Compared with the control group, the patients in the E-coach group had better drug compliance following the intervention (6.8 ± 1.9 vs. 7.9 ± 1.0), and the difference was statistically significant (*p* < 0.05). There was no significant difference in ABI between the two groups before the operation, 1 week after the operation or 6 months after the operation (*p* < 0.05) (Tables 5 and 6).

**Table 5:** Comparison of drug compliance between the two groups before and after intervention

Group	*n*	Before intervention [*n* (%)]			After intervention [*n* (%)]			Before intervention	After intervention	Score difference
		Good	Medium	Poor	Good	Medium	Poor	$\overline{x} \pm s$	$\overline{x} \pm s$	$\overline{x} \pm s$
Control group	96	32 (33.3)	36 (37.5)	28 (29.2)	41 (33.3)	36 (37.5)	19 (29.2)	5.3 ± 1.5	6.8 ± 1.9	1.5 ± 1.2
E-coach group	108	36 (33.3)	35 (32.4)	37 (34.3)	60 (55.6)	43 (39.8)	5 (4.6)	5.6 ± 0.9	7.9 ± 1.0	2.3 ± 1.7
*t*/*χ*^2^		0.419			− 3.306			0.352	5.369	7.587^2)^
*P*		0.573			0.002			0.771	< 0.001	0.000

**Table 6:** Comparison of ABI between the two groups before and after intervention

Group	*n*	Before operation	1 week after operation	6 months after operation
Control group	96	0.28 ± 0.10	0.61 ± 0.13	0.90 ± 0.15
E-coach group	108	0.29 ± 0.15	0.64 ± 0.17	1.02 ± 0.13
*t*		0.528	2.146	0.472
*P*		0.596	0.321	0.637

### Comparison of six-minute walking and pain-free walking distance test results before and after intervention across the two groups

There were no significant differences in the 6MWT and PFWD scores between the two groups before the intervention (*p* > 0.05). The 6MWT scores (329.19 ± 5.58 vs. 353.00 ± 9.76; 412.65 ± 12.59 vs. 499.16 ± 18.43) and PFWD scores (219.15 ± 11.96 vs. 225.36 ± 16.13; 331.62 ± 51.36 vs. 369.42 ± 75.71) were significantly higher in the E-coach group than in the control group at 1 and 6 months after the intervention (*p* < 0.05) (Table 7).

**Table 7:** Comparison of 6MWD and PFWD before and after intervention in two groups

Group	*n*	6MWD			PFWD		
		Before intervention	1 month after intervention	6 months after intervention	Before intervention	1 month after intervention	6 months after intervention
Control group	96	309.98 ± 1.98	329.19 ± 5.58	412.65 ± 12.59	149.06 ± 15.15	219.15 ± 11.96	331.62 ± 51.36
E-coach group	108	301.65 ± 2.59	353.00 ± 9.76	499.16 ± 18.43	151.33 ± 11.25	225.36 ± 16.13	369.42 ± 75.71
*t*		− 1.431	− 3.331	− 7.437	− 1.165	− 3.015	− 7.371
*P*		0.115	0.001	< 0.001	0.148	0.001	< 0.001

## Discussion

### Innovative features of the E-coach chronic disease management model

The main characteristic of the E-coach chronic disease management mode is the optimisation and integration of internet and traditional health coaching technology, which gives full play to the strengths of each *and* compensates for their shortcomings. Studies at home and abroad have shown that the Internet-based E-coach chronic disease management can achieve the purpose of 30-day readmission rate and control of hypertension in patients with chronic obstructive pulmonary disease [8, 15]. In the early stages of this study, the expert group agreed that the integration and optimisation of internet technology and health coaching can maximise the effect of chronic disease management and reduce the cost of both the management and the medical insurance. The application of the E-coach chronic disease management model in the endovascular treatment of patients with ASO further confirmed the effectiveness of this model in chronic disease management. The main reasons include establishing good communication channels with patients, formulating rehabilitation programmes that meet the support needs of patients and their families, increasing patients’ sense of achievement, integrating health goals into daily life, and ensuring the effectiveness, feasibility and sustainability of measures [16].

### *Improving the walking ability and limb function of patients after arteriosclerosis obliterans intervention *via* E-coach chronic disease management model supervisory exercise training*

The recovery and prognosis of lower limb function are the most important problems in patients with ASO, and exercise is closely associated with the recovery of this function. The results of this study showed that the scores of 6MWT and PFWD in both groups increased with time, and the scores of E-coach group increased significantly. Many studies have shown that postoperative guidance of Buerger exercise is effective in improving lower limb symptoms and walking ability [17–19], and elastic band lower limb resistance training is effective in improving patients’ physical function and muscle strength [20]. The results of this study confirm this. In this study, Buerger exercise, fast walking training + Buerger exercise, and lower limb resistance training + fast walking training + Buerger exercise programme step-by-step intensive training, combined with an emphasis on drug compliance, lifestyle, and risk factor intervention, encouraged the patients to implement health plans, helped with observing and confirming the patients’ health status and addressing problems according to the patient data detection trend charts, and ensured the effectiveness and feasibility of the measures. In this study, the E-coach mode-supervised sports training, individualised sports programmes, and full-course supervision, alongside the guidance of the researchers, had significant effects on increasing the patients’ walking distance and improving their limb function.

### Improvement in the self-management ability of patients after arteriosclerosis obliterans intervention using the E-coach chronic disease management model

Kang’s research showed that health guidance and electronic health management programmes can effectively improve patients’ self-efficacy [21]. This study inherits the knowledge of disease management, exercise rehabilitation, and the importance of medication, realizes the subtle educational effect, enhances the patients’ understanding of the importance of rehabilitation management, helps them understand the importance of rehabilitation training, improves self-confidence, and enhances the awareness of independent exercise. In the process of rehabilitation, patients actively seek professional help from medical staff. Under the remote supervision and reminder of researchers, patients and their families can actively participate in the activities, which effectively improves the patient’s medication compliance. The effect on blood pressure, fasting blood glucose, and compliance rate was significantly better than that of the control group.

### Limitations

There are still some limitations in this study. The sample size of this study is small. Promoting this model and ensuring its effective operation is the top priority of this study. The next step, the research group will take community linkage, a variety of ways to promote health management activities.

## Conclusion

The E-coach chronic disease management model can effectively improve the blood glucose and blood pressure control rates and the behaviour management of patients with ASO and is thus worthy of clinical reference.
